# Association of Heavy Metals with Overall Mortality in a Taiwanese Population

**DOI:** 10.3390/nu13062070

**Published:** 2021-06-17

**Authors:** Yi-Hsueh Liu, Chih-Wen Wang, Da-Wei Wu, Wen-Hsien Lee, Ying-Chih Chen, Chiu-Hui Li, Chun-Chi Tsai, Wen-Yi Lin, Szu-Chia Chen, Chih-Hsing Hung, Chao-Hung Kuo, Ho-Ming Su

**Affiliations:** 1 Department of Internal Medicine, Kaohsiung Municipal Siaogang Hospital, Kaohsiung Medical University, Kaohsiung 812, Taiwan; liuboy17@gmail.com (Y.-H.L.); chinwin.wang@gmail.com (C.-W.W.); u8900030@yahoo.com.tw (D.-W.W.); cooky-kmu@yahoo.com.tw (W.-H.L.); 990329kmuh@gmail.com (Y.-C.C.); scarchenone@yahoo.com.tw (S.-C.C.); kjh88kmu@gmail.com (C.-H.K.); 2 Graduate Institute of Clinical Medicine, College of Medicine, Kaohsiung Medical University, Kaohsiung 812, Taiwan; 3 Division of Cardiology, Department of Internal Medicine, Kaohsiung Medical University Hospital, Kaohsiung Medical University, Kaohsiung 812, Taiwan; 4 Faculty of Medicine, College of Medicine, Kaohsiung Medical University, Kaohsiung 812, Taiwan; 5 Health Management and Occupational Safety Health Center, Kaohsiung Municipal Siaogang Hospital, Kaohsiung Medical University, Kaohsiung 812, Taiwan; 996117@kmsh.org.tw (C.-H.L.); 1036124@kmuh.org.tw (C.-C.T.); 810064@kmuh.org.tw (W.-Y.L.); 6 Research Center for Environmental Medicine, Kaohsiung Medical University, Kaohsiung 812, Taiwan; pedhung@gmail.com

**Keywords:** heavy metals, mortality, Taiwanese Population, cadmium, copper, manganese

## Abstract

Previous studies have shown links between heavy metals and many health issues. However, data on the association between heavy metals and mortality in the general population are still limited. Therefore, the aim of this study was to investigate the relationship between heavy metals and overall mortality in the general population. We enrolled 2497 participants (1001 males and 1496 females) living in southern Taiwan, and measured levels of seven heavy metals: lead (Pb) in blood and cadmium (Cd), nickel (Ni), copper (Cu), chromium (Cr), manganese (Mn) and arsenic (As) in urine. The median follow-up period was 41.8 (4–50) months, during which 40 (1.6%) patients died. Compared to the participants who survived, those who died had higher urine Cd, higher urine Cu and lower urine Mn levels. Multivariate analysis showed that high urine Cd (per 1 μg/L; hazard ratio [HR], 1.352; 95% confidence interval [CI], 1.089–1.680; *p* = 0.006), high urine Cu (per 1 μg/dL; HR, 1.350; 95% CI, 1.151–1.583; *p* < 0.001), and low urine Mn (per 1 μg/L; HR, 0.717; 95% CI, 0.557–0.923; *p* = 0.010) were associated with increased overall mortality. In conclusion, our results demonstrated that high levels of urine Cd and Cu and low urine Mn level were associated with increased overall mortality in the general population.

## 1. Introduction

Heavy metals are defined as metallic elements that have a relatively high density compared to water. Some metals such as zinc, copper (Cu), iron, manganese (Mn), and cobalt are required for various biochemical and physiological functions in humans at low concentrations, but they become toxic at higher concentrations. Other heavy metals such as lead (Pb), mercury, and cadmium (Cd) are not known to have any beneficial effects on human health and their accumulation in the human body is deleterious to human health [1]. Heavy metals are common environmental contaminants and toxicity due to heavy metals is a growing concern [2]. Heavy metals can disrupt metabolic functions through various mechanisms and their toxicity depends on factors such as dose, chemical form, and route of exposure, in addition to age, sex, and nutritional and genetic status of the exposed individual [3]. Moreover, heavy metals can accumulate in organs such as liver, heart, kidneys, and brain and disrupt normal biological functions.

Some heavy metals such as arsenic (As), Cd, chromium (Cr), Pb, and mercury, are systemic toxicants, which have been shown to cause adverse health effects in humans, including developmental abnormalities, cardiovascular diseases, renal injury, neurologic and neurobehavioral disorders, diabetes, hematologic and immunologic disorders, and various types of cancer [3]. Heavy metal-induced toxicity has been shown to be through the production of reactive oxygen species, resulting in oxidative damage and adverse effects on health [1]. Previous studies have reported associations between mortality and various heavy metals. For example, Pb exposure has been shown to be a risk factor for cardiovascular mortality and even a low blood Pb concentration has been associated with increased risks of all-cause mortality, cardiovascular disease, and cancer [4,5,6,7]. In addition, exposure to low concentrations of Cd has also been associated with increased risks of overall and cardiovascular mortality [8,9,10,11]. For nickel (Ni), increased rates of mortality and cancer have been reported in Ni workers and people living in highly polluted environments [12,13,14,15]. Chronic As exposure through contaminated drinking water has also been associated with increase all-cause and chronic disease-related mortality [16,17]. In addition, several studies have reported potential associations between high serum Cu concentrations and increased overall, cancer, and cardiovascular-related mortality [18,19,20,21].

Although the effects of heavy metals on human health have been widely discussed in the literature, studies on the effect of heavy metal concentrations within relative normal limits on mortality in the general population are still limited. Therefore, the aim of this study was to investigate the relationships between heavy metals, including Pb in blood, and Cd, Ni, Cu, Cr, Mn, and as in urine with overall mortality in people living in southern Taiwan.

## 2. Materials and Methods

### 2.1. Subject Recruitment 

We conducted a health survey from June 2016 to September 2018 in southern Taiwan, which was promoted thorough advertisements. All subjects who attended the health survey and were willing to participate in this study were enrolled. Subsequently, they all underwent physical examinations and an experienced physician recorded their clinical histories. Anthropometric measurements including systolic blood pressure, diastolic blood pressure, and weight and height were taken. 

### 2.2. Ethics Statement 

The study protocol was approved by the Institutional Review Board of Kaohsiung Medical University Hospital (number: KMUHIRB-G(II)-20190011). All participants provided informed consent before study enrollment.

### 2.3. Collection of Demographic, Medical, and Laboratory Data 

In addition to the anthropometric measurements, the following variables were recorded at baseline: demographics (age and sex), medical history (diabetes mellitus [DM], hypertension, hyperlipidemia, and laboratory data (fasting glucose, serum creatinine, triglycerides, total cholesterol, high- [HDL]- and low-density lipoprotein [LDL]-cholesterol). Body mass index (BMI) was calculated as weight/height squared (kg/m^2^).

### 2.4. Measurement of Blood and Urine Heavy Metal Concentrations 

Seven heavy metals were measured: The concentrations of Pb in blood was analyzed using graphite furnace atomic absorption spectrometry (AA800, Perkin Elmer), and Cd, Ni, Cu, Cr, Mn, and As in urine were analyzed using inductively coupled plasma mass spectrometry (ICP-MS, NexION 300 Series, Perkin Elmer). Details of the instrumental analysis have been reported by a CAP (COLLEGE of AMERICAN PATHOLOGISTS) accredited clinical laboratory (Union clinical laboratory Taipei, Taiwan, CAP Number: 6979606). The capability of tests is monitored through regular routine daily internal quality control testing and external proficiency test.

### 2.5. Definition of Overall Mortality

Data on overall mortality were collected from the Collaboration Center of Health Information Application (CCHIA), Ministry of Health and Welfare, Executive Yuan, Taiwan, up to July 2020. The follow up period was from the date of enrollment to death or July 2020.

### 2.6. Statistical Analysis

Statistical analysis was performed using SPSS version 22.0 for Windows (SPSS Inc., Chicago, IL, USA). Data were expressed as percentages, means ± standard deviations, or medians (25th–75th percentiles) for heavy metals. Between-group differences in categorical variables were analyzed using the chi-square test and between-group differences in continuous variables were analyzed using the independent *t*-test. Multivariate Cox proportional hazard analysis was used to identify associations between the heavy metals and overall mortality. Significant variables in univariate analysis were entered into the multivariable analysis. A *p* value of less than 0.05 was considered to indicate a statistically significant difference.

## 3. Results 

The mean age of the 2497 participants (1001 males and 1496 females) was 54.3 ± 14.0 years. A comparison of the clinical characteristics between the survivors and non-survivors is shown in Table 1. Compared to the survivors, the non-survivors were older and had higher prevalence rates of DM and hypertension, a higher level of creatinine, and lower level of LDL-cholesterol. In addition, the non-survivors had higher urinary concentrations of Cd and Cu and a lower urinary concentration of Mn.

### Predictors of Overall Mortality 

The median follow-up period was 41.8 (range 4–50) months for all participants, during which 40 (1.6%) patients died. Table 2 shows the associations among demographic, medical, laboratory, and heavy metal data with overall mortality. In the univariate analysis, old age (per 1 year; hazard ratio [HR], 1.097; 95% confidence interval [CI], 1.066–1.128; *p* < 0.001), DM (HR, 4.049; 95% CI, 2.089–7.848; *p* < 0.001), hypertension (HR, 3.478; 95% CI, 1.865–6.485; *p* < 0.001), high serum creatinine (per 1 mg/dL; HR, 1.998; 95% CI, 1.482–2.695; *p* < 0.001), low LDL-cholesterol (per 1 mg/dL; HR, 0.990; 95% CI, 0.980–0.999; *p* = 0.037), high urine Cd (per 1 μg/L; HR, 1.290; 95% CI, 1.070–1.554; *p* = 0.008), high urine Cu (per 1 μg/dL; HR, 1.378; 95% CI, 1.241–1.549; *p* < 0.001), and low urine Mn (per 1 μg/L; HR, 0.726; 95% CI, 0.567–0.930; *p* = 0.011) were associated with increased overall mortality.

In the multivariate Cox proportional hazard analysis, after adjusting for age, DM, hypertension, serum creatinine, and LDL-cholesterol, high urine Cd (per 1 μg/L; HR, 1.352; 95% CI, 1.089–1.680; *p* = 0.006), high urine Cu (per 1 μg/dL; HR, 1.350; 95% CI, 1.151–1.583; *p* < 0.001), and low urine Mn (per 1 μg/L; HR, 0.717; 95% CI, 0.557–0.923; *p* = 0.010) were significantly associated with increased overall mortality (Table 3).

## 4. Discussion

In this study, we evaluated the relationships between heavy metals and overall mortality in 2497 participants living in southern Taiwan and found that high urine Cd, high urine Cu, and low urine Mn were associated with increased overall mortality. 

The first important finding of this study is that high urine Cd was associated with increased overall mortality. Cd is a non-essential nutrient and an extremely toxic industrial and environmental pollutant which is classified as a human carcinogen [22]. Cigarette smoke is a major source of Cd exposure and food is the major source of Cd exposure in non-smokers [23]. Cd compounds are mainly used in re-chargeable Ni-Cd batteries and emissions of Cd have increased substantially in the 20th century [24]. As urinary Cd excretion is slow, Cd accumulates in the body, particularly in the kidneys and liver, with a reported biological half-life of 10–30 years [22]. Chronic Cd toxicity has been associated with renal tubular dysfunction, reduced bone mineral density, normochromic anemia, and some cancers [25]. In the present study, we found that high urine Cd was associated with increased overall mortality, which is consistent with previous studies [8,9,10,11,26]. There is no margin of safety between exposure and the point of departure in the general population, and therefore it is important to limit exposure to Cd as far as possible [27].

The second important finding of this study is that high urine Cu was correlated with increased overall mortality. Cu is common in the environment and an essential nutrient. Cu acts as a critical cofactor and plays important roles in many metalloenzymes involved in drug metabolism, hemoglobin formation, pigment formation, cellular respiration, iron oxidation, carbohydrate metabolism, catecholamine biosynthesis, connective tissue formation, and antioxidant defense mechanisms [28,29]. The most common routes of exposure to Cu in humans are through inhalation, oral intake, and dermal contact, but mainly through food. Cu is taken up by the liver and excreted in bile or secreted into plasma [30]. The most sensitive adverse effect of excess exposure to Cu is gastrointestinal tract irritation, causing acute nausea, vomiting, and diarrhea [31]. At higher doses, Cu toxicity can cause headache, gastrointestinal hemorrhage, hemolytic anemia, and liver and kidney failure [32]. Cu is not known to be carcinogenic or mutagenic or to have reproductive or in utero effects [31]. On the other hand, Cu deficiency can cause anemia, leucopenia, and myeloneuropathy [33]. In the present study, high urine Cu was associated with an increase in mortality, which is consistent with previous studies [18,19,20,21]. The mechanism for this increase in mortality is unclear, however, it may be related to increased inflammation, oxidative stress, or the metabolic syndrome [34,35]. High concentrations of Cu have been shown to potentially cause oxidative damage to proteins, lipids, and DNA and also to play a role in neurodegenerative disorders [30].

Another important finding of this study is that low urine Mn was associated with increased overall mortality. Mn is an essential nutrient that plays a role in growth and tissue formation, metabolism, and reproduction [36,37]. Mn is mainly absorbed through the gastrointestinal tract, but also through inhalation and intravenous routes [38]. Mn is primarily eliminated via fecal hepatobiliary excretion and to a lesser extent urinary excretion. Mn deficiency is rare and is related to reduced fertility, deficient bone formation, impaired growth, skeletal defects, birth defects, altered lipid and carbohydrate metabolism, and abnormal glucose tolerance in both animals and humans [39]. Mn is a cofactor in various enzymes and plays crucial roles in reproduction, development, digestion, energy production, antioxidant defense, regulation of neuronal activities, and immune responses [36], which may partially explain our present finding, i.e., low urine Mn was associated with increased overall mortality.

There are several limitations to this study. Firstly, the follow-up period was relatively short, and a longer follow-up period may provide a better understanding of the associations between heavy metal concentrations and mortality. Nevertheless, our findings highlight the important link between heavy metals and mortality. Secondly, only 40 deaths were reported among 2497 participants, and low study power must be considered in this study. Thirdly, potential selection bias should be considered because the cohort was enrolled through advertisement. The volunteers were unlikely to be representative of the background population. In addition, the study population was totally selected from southern Taiwan, an industrial area, might have a relatively high risk of exposure to heavy metals. Thus, there must be caution about our findings when applied to the general population. Fourthly, we could not identify the sources or routes of heavy metal exposure in our study cohort. Even though we measured blood and urinary concentrations of the heavy metals and obtained serum biochemical data, different exposure methods and routes may have different effects. Finally, we measured total As concentrations but could not measure the level of inorganic As in urine due to limitations in our equipment, and this may have led to an underestimation of the contribution of As on mortality.

In conclusion, our results demonstrated that in the general population, high urine Cd and Cu and low urine Mn were associated with increased overall mortality. 

## Figures and Tables

**Table 1:** Comparison of clinical characteristics between the survivors and non-survivors.

Characteristics	All (*n* = 2497)	Survival (*n* = 2457)	Death (*n* = 40)	*p*
Age (year)	54.3 ± 14.0	54.1 ± 14.0	69.1 ± 11.4	<0.001
Male gender (%)	40.1	40.0	45.0	0.523
DM (%)	10.3	9.9	32.5	0.005
Hypertension (%)	24.8	24.3	55.0	<0.001
Hyperlipidemia (%)	2.2	2.2	2.5	0.912
BMI (kg/m^2^)	25.0 ± 3.9	25.0 ± 3.9	25.2 ± 4.5	0.834
SBP (mmHg)	131.8 ± 19.8	131.7 ± 19.7	137.2 ± 21.3	0.083
DBP (mmHg)	77.4 ± 11.7	77.4 ± 11.7	78.1 ± 11.6	0.691
Laboratory parameters				
Fasting glucose (mg/dL)	99.6 ± 27.2	99.5 ± 27.1	106.9 ± 31.7	0.087
Serum Creatinine (mg/dL)	0.95 ± 0.29	0.94 ± 0.28	1.18 ± 0.53	0.007
Triglyceride (mg/dL)	126.1 ± 97.4	126.0 ± 97.3	138.2 ± 100.2	0.428
Total cholesterol (mg/dL)	199.2 ± 37.5	199.3 ± 37.4	191.4 ± 43.3	0.186
HDL-cholesterol (mg/dL)	53.0 ± 13.6	53.0 ± 13.6	50.5 ± 15.4	0.237
LDL-cholesterol (mg/dL)	118.9 ± 34.0	119.1 ± 33.9	108.4 ± 36.1	0.048
Blood				
Pb (μg/dL)	1.5 (1.0–2.2)	1.8 (1.0–2.2)	2.1 (1.3–2.8)	0.201
Urine				
Cd (μg/L)	0.8 (0.3–1.4)	1.1 (0.2–1.4)	1.5 (0.5–2.4)	0.017
Ni (μg/L)	2.5 (1.5–3.7)	3.3 (1.5–3.7)	3.8 (1.9–5.4)	0.829
Cu (μg/dL)	1.5 (1.0–2.0)	1.6 (1.0–2.0)	2.55 (1.5–3.6)	<0.001
Cr (μg/L)	0.1 (0.1–0.1)	0.1 (0.1–0.1)	0.1 (0.1–0.1)	0.787
Mn (μg/L)	1.7 (0.9–3.0)	2.2 (0.9–3.0)	1.5 (0.6–2.0)	0.019
As (μg/L)	77.9 (44.5–140.2)	118.2 (44.0–139.4)	159.9 (69.9–205.4)	0.064

Abbreviations. DM, diabetes mellitus; BMI, body mass index; SBP, systolic blood pressure; DBP, diastolic blood pressure; HDL, high-density lipoprotein; LDL, low-density lipoprotein; Pb, lead; Cd, cadmium; Ni, nickel; Cu, copper; Cr, chromium; Mn, manganese; As, arsenic.

**Table 2:** Predictors of overall mortality using Cox proportional hazards model.

Parameters	Univariable Analysis	
	HR (95% CI)	*p*
Age (per 1 year)	1.097 (1.066–1.128)	<0.001
Male (vs. female)	1.259 (0.675–2.347)	0.469
DM	4.049 (2.089–7.848)	<0.001
Hypertension	3.478 (1.865–6.485)	<0.001
Hyperlipidemia	1.193 (0.164–8.685)	0.862
BMI (per 1 kg/m^2^)	1.007 (0.929–1.090)	0.873
SBP (per 1 mmHg)	1.012 (0.998–1.027)	0.090
DBP (per 1 mmHg)	1.007 (0.981–1.033)	0.615
Labortaory data		
Fasting glucose (per 1 mg/dL)	1.006 (0.998–1.014)	0.141
Creatinine (per 1 mg/dL)	1.998 (1.482–2.695)	<0.001
Total cholesterol (per 1 mg/dl)	0.994 (0.985–1.002)	0.155
Triglyceride (per 1 mg/dL)	1.001 (0.999–1.003)	0.366
HDL-cholesterol (per 1 mg/dL)	0.986 (0.962–1.010)	0.254
LDL-cholesterol (per 1 mg/dL)	0.990 (0.980–0.999)	0.037
Blood		
Pb (per 1 μg/dL)	1.067 (0.931–1.222)	0.350
Urine		
Cd (per 1 μg/L)	1.290 (1.070–1.554)	0.008
Ni (per 1 μg/L)	1.001 (0.989–1.013)	0.866
Cu (per 1 μg/dL	1.378 (1.241–1.529)	<0.001
Cr (per 1 μg/L)	1.101 (0.534–2.268)	0.795
Mn (per 1 μg/L)	0.726 (0.567–0.930)	0.011
As (per 1 μg/L)	1.001 (1.000–1.003)	0.127

Values expressed as hazard ratio (HR) and 95% confidence interval (CI). Abbreviations are the same as in Table 1.

**Table 3:** Association of heavy metals with overall mortality using multivariate Cox proportional hazards model.

Heavy Metals	Multivariable	
	HR (95% CI)	*p*
Blood		
Pb (per 1 μg/dL)	1.024 (0.865–1.231)	0.780
Urine		
Cd (per 1 μg/L)	1.352 (1.089–1.680)	0.006
Ni (per 1 μg/L)	1.004 (0.990–1.018)	0.599
Cu (per 1 μg/dL)	1.350 (1.151–1.583)	<0.001
Cr (per 1 μg/L)	1.015 (0.513–2.009)	0.966
Mn (per 1 μg/L)	0.717 (0.557–0.923)	0.010
As (per 1 μg/L)	1.001 (0.999–1.003)	0.424

Values expressed as hazard ratio (HR) and 95% confidence interval (CI). Abbreviations are the same as in Table 1. Covariates in the multivariable model included age, sex, DM, hypertension, creatinine, and LDL-cholesterol.

## Data Availability

The datasets used and analyzed during the current study are available from the corresponding author on reasonable request.
